# An Occult Primary Thymic Mucosa‐Associated Lymphoid Tissue Lymphoma With Sjögren's Syndrome Revealed by CXCR4 Targeted ^68^Ga‐Pentixafor PET/CT

**DOI:** 10.1111/jcmm.70248

**Published:** 2025-01-31

**Authors:** Xuehan Gao, Xingtong Peng, Rongxi Wang, Zhihong Qian, Xiaoyun Zhou, Zhaohui Zhu, Yeye Chen

**Affiliations:** ^1^ Department of Thoracic Surgery Peking Union Medical College Hospital, Chinese Academy of Medical Sciences & Peking Union Medical College Beijing China; ^2^ Department of Nuclear Medicine, State Key Laboratory of Complex Severe and Rare Diseases, Beijing Key Laboratory of Molecular Targeted Diagnosis and Therapy in Nuclear Medicine Peking Union Medical College Hospital, Chinese Academy of Medical Sciences, Peking Union Medical College Beijing China; ^3^ Department of Basic Medical Sciences Chinese Academy of Medical Sciences and Peking Union Medical College Beijing China

**Keywords:** ^68^Ga‐pentixafor, mucosa‐associated lymphoid tissue lymphoma, PET/CT, Sjögren's syndrome

## Abstract

Mucosa‐associated lymphoid tissue (MALT) lymphoma is an extranodal low‐grade non‐Hodgkin lymphoma that extremely rarely localises to the mediastinum. A 34‐year‐old female with chronic arthralgia, sicca and rash was found to have a well‐demarcated mediastinal cystic mass with equivocal nodular enhancement within the cystic wall on chest CT during a workup for Sjögren's syndrome. Subsequent ^68^Ga‐Pentixafor‐PET/CT revealed focal uptake increase within the cystic capsule. The patient underwent thoracoscopic resection of the mediastinal lesion, and pathology revealed MALT lymphoma in the wall of a thymic cyst. This case highlights that ^68^Ga‐pentixafor PET/CT could be valuable for the non‐invasive detection of occult thymic MALT lymphoma.

**Trial Registration:** ClinicalTrials.gov. (www.clinicaltrials.gov, NCT06086327)

## 1 Clinical History

**FIGURE 1:**
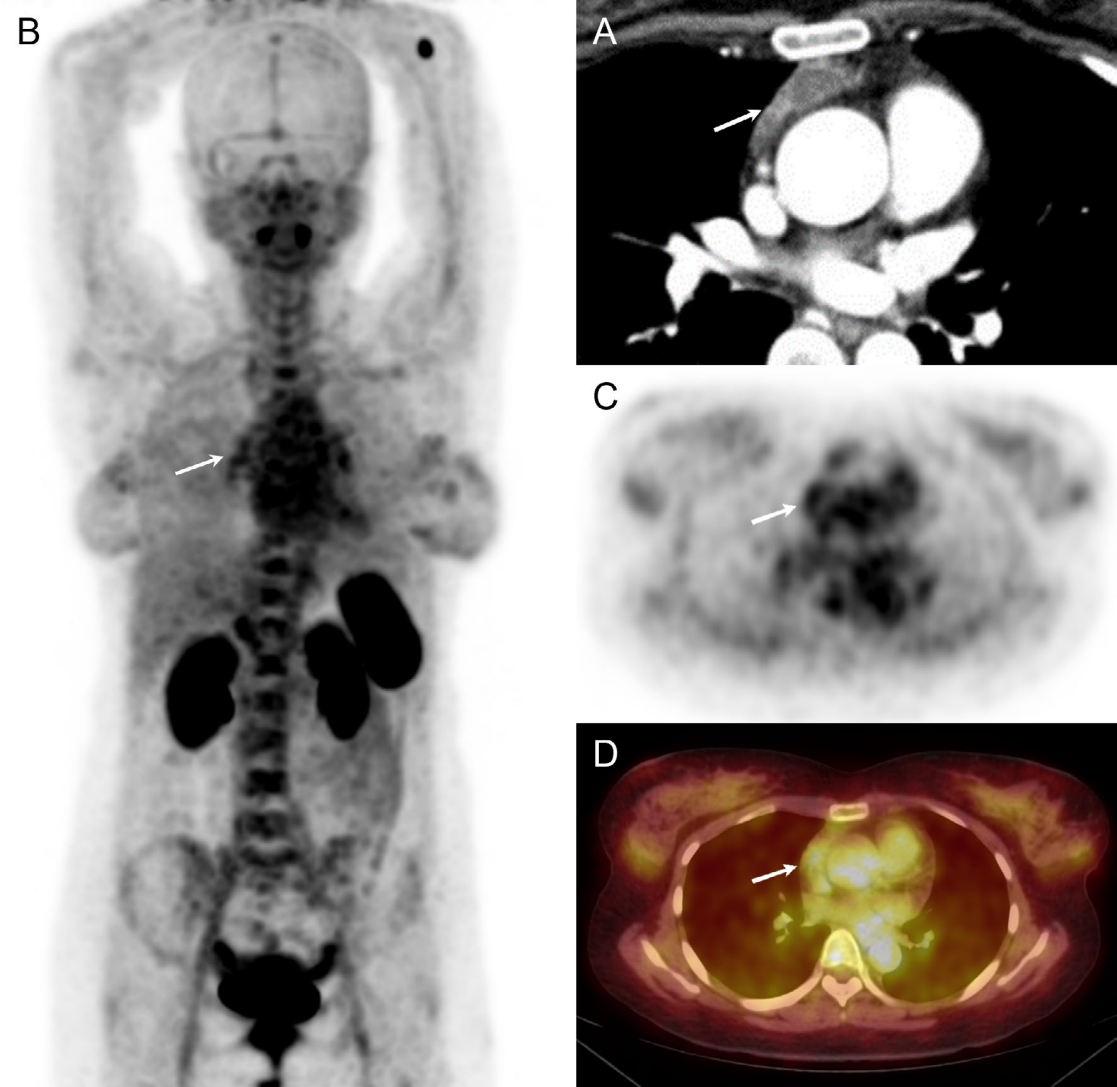
^68^Ga‐Pentixafor PET/CT scans of a thymic cyst MALT lymphoma. Contrast‐enhanced chest CT showing a well‐demarcated mediastinal cyst with mildly enhancing focal thickening of the cystic wall (A). Maximum intensity projections (MIPs) (B), PET axial view (C) and PET/CT fusion (D) of ^68^Ga‐Pentixafor PET/CT showing a thymic cystic MALT lymphoma with mild uptake.

A 34‐year‐old female with chronic dry eyes and mouth, intermittent arthralgia and skin purpura for 6 years presented to our outpatient clinic. The patient also had a mediastinal cystic occupation that was found incidentally 1 year ago, of unknown clinical significance. A diagnosis of Sjögren's syndrome was made following pertinent evaluation and the mediastinal cystic occupation was subjected to further investigation to rule out thymoma, which could lead to autoimmune disease. Contrast‐enhanced chest CT revealed a well‐demarcated, mediastinal cyst with mildly enhancing focal thickening of the cystic wall (Figure 1A, white arrow).

## 2 Results

**FIGURE 2:**
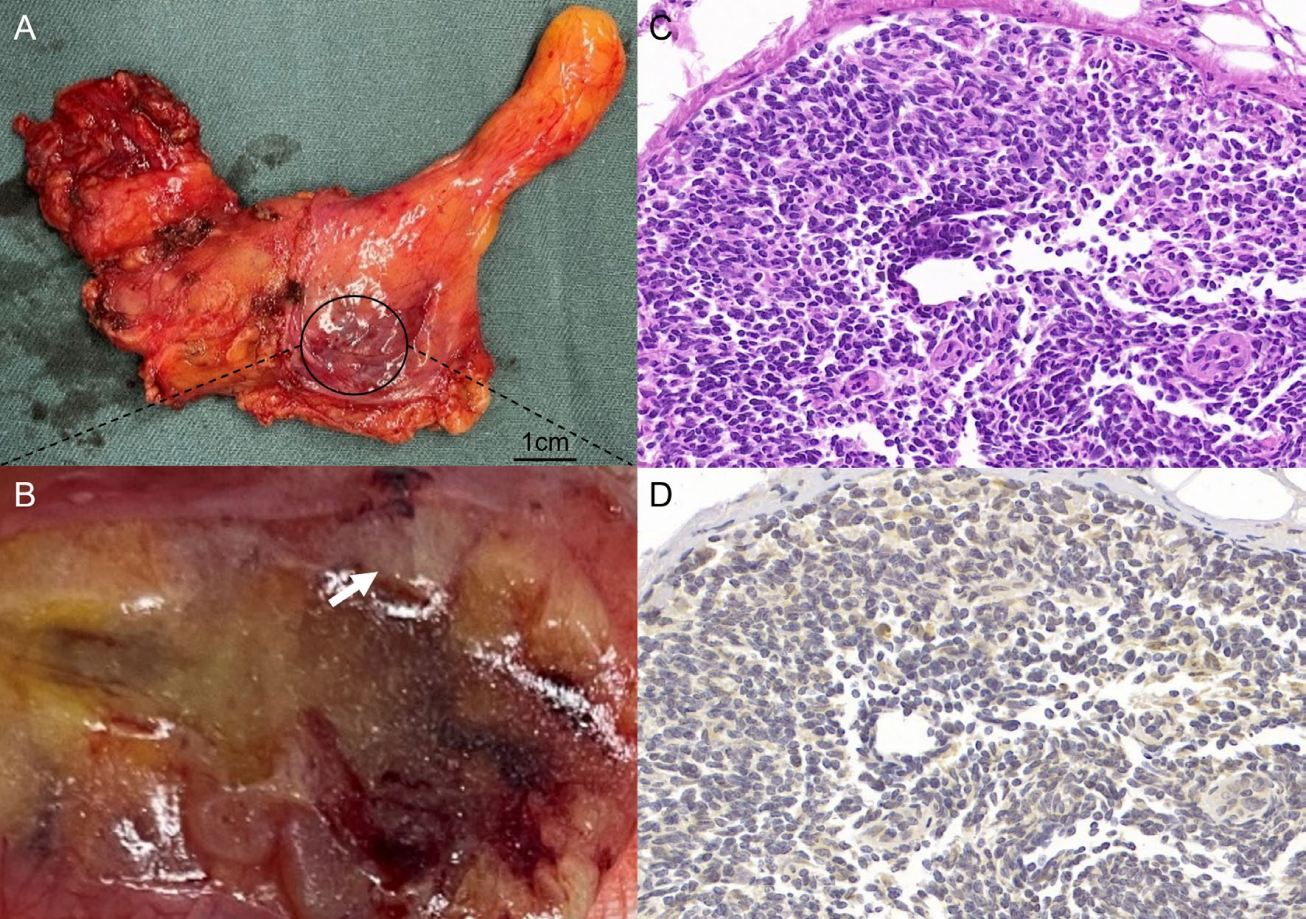
Macroscopic findings (A) and the cut surface (B) of thymic cyst MALT lymphoma. Haematoxylin and eosin staining (40× magnification) showing a MALT lymphoma (C). Immunohistochemistry staining (40× magnification) showing expression of CXCR4 in MALT lymphoma (D).

To help better characterise the mediastinal lesion, the patient was enrolled in our ^68^Ga‐Pentixafor PET/CT study (NCT06086327) with appropriate informed consent. ^68^Ga‐pentixafor is a novel radioligand specifically targeting C‐X‐C chemokine receptor type 4 (CXCR4) that has been implicated in detection of various hematologic and solid malignancies [1, 2]. Focal radioactive uptake (SUVmax, 2.95; white arrow) was noted within the cystic capsule (Figure 1B, MIP; Figure 1C, PET axial view; Figure 1D, PET/CT fusion) by ^68^Ga‐Pentixafor PET/CT, raising the suspicion for an occult neoplasm, indicating an occult lower grade tumour such as haematological neoplasms. Subsequently, the patient underwent video‐assisted thoracoscopic resection of anterior mediastina mass, and a cystic lesion was observed intraoperatively. On macroscopic examination, the cystic lesion was located within a grossly thymus looking tissue and measured approximately 3 × 3 cm (Figure 2A). The cystic wall was locally thickened that measured about 1 × 1 cm (Figure 2B, white arrow). Pathology revealed hyperplasia of lymphocytic tissue infiltrating normal thymic tissue (Figure 2C, Haematoxylin and eosin staining, 40× magnification). Immunohistochemical staining revealed that the lesion was diffusely and strongly positive for Bcl‐2, and scattered positivity for Bcl‐6, CD20, CD21 and CD3. The Ki67 proliferation index was 30%. Additional immunohistochemistry staining demonstrated expression of CXCR4 within the tumour (Figure 2D), in concordance to ^68^Ga‐pentixafor PET/CT findings.

## 3 Diagnosis

The pathological examination and immunohistochemical results indicated a final diagnosis of a mucosa‐associated lymphoid tissue (MALT) lymphoma with thymic cyst.

## 4 Discussion

MALT lymphoma is an extranodal, low‐grade non‐Hodgkin lymphoma (NHL) and accounts for 5%–8% of all NHLs, which can present at any extranodal sites. Gastrointestinal tract is the most common site of NHLs, followed by orbit and lung, and rarely in thymus [3]. Isaacson and Wright et al. first described MALT lymphoma as a new subtype of extranodal malignant lymphoma of marginal zone B‐cell origin in 1983 [4]. According to the fifth edition of the WHO Classification of the Haematopoietic and Lymphoid Tissue [5] and the updated International Consensus Classification of Mature Lymphoid Neoplasm [6], MALT lymphoma is currently recognised as a clinicopathologic entity of MZL.

MALT lymphoma is closely associated with various autoimmune processes, especially Sjögren's syndrome [7, 8, 9], but the related mechanism has not yet been fully revealed. Primary thymic MALT lymphoma, originating from the mucosal tissue of the thymus, is extremely rare and more prevalent in Asian populations, primarily affecting females. Thymic MALT lymphoma lacks specific clinical manifestations and commonly presents as a non‐special multicystic mediastinal mass in chest CT, mimicking thymoma, mediastinal seminoma and non‐neoplastic thymic lesions. Histopathological examination of biopsy specimens is the gold standard for diagnosing MALT lymphoma.

CXCR4 is a member of the G protein‐coupled receptors mainly expressed in stem cells, haematopoietic cells and various immune cells, and has been implicated in multiple tumorigenic pathways. Almost all lymphocytes, including MZL cells, express CXCR4 physiologically. CXCR4‐targeting ^68^Ga‐pentixafor PET/CT is a novel PET tracer and has demonstrated suitability for identifying and evaluating various types of hematologic and solid neoplasms. Previous studies have indicated the superiority of ^68^Ga‐pentixafor in detecting MALT lymphoma compared to ^18^F‐FDG [10, 11]. Johannes Duell et al. demonstrated that ^68^Ga‐pentixafor PET/CT could accurately identify all MZL patients and is more suitable for early staging of MZL compared to conventional staging methods [11]. The proliferative activity indicated by the Ki‐67 index of MZL lesions correlated positively with the intensity of ^68^Ga‐pentixafor imaging signals. Immunohistochemical staining revealed moderate expression of CXCR4 receptors on the surface of MZL cells. However, previous studies focused on common sites of MALT lymphoma, such as the orbit, gastrointestinal tract, lung, salivary gland and cervical lymph nodes. There have been no published reports in the diagnosis of primary thymic MALT lymphoma using ^68^Ga‐pentixafor. As primary thymic MALT lymphoma is rare and lacks specific clinical features, it may be easily misdiagnosed as thymoma or other thymic benign lesions [12]. Additionally, percutaneous aspiration is usually inadequate for diagnosing thymic MALT lymphoma due to the risk of haemorrhage, needle tract metastasis and seeding if the lesion is malignant. Therefore, a new feasible and non‐invasive method for identifying thymic MALT is needed.

The present case highlights the importance of considering primary thymic MALT lymphoma in patients presenting with a thymic cystic lesion, particularly when complicated by Sjögren's syndrome. Additionally, ^68^Ga‐pentixafor PET/CT has been shown to be an effective non‐invasive tool for diagnosing occult thymic MALT lymphoma.

## Author Contributions

**Xuehan Gao:** conceptualization (equal), data curation (equal), formal analysis (equal), investigation (equal), methodology (equal), writing – original draft (lead), writing – review and editing (equal). **Xingtong Peng:** conceptualization (equal), data curation (equal), formal analysis (equal), investigation (equal), methodology (equal), writing – original draft (supporting), writing – review and editing (supporting). **Rongxi Wang:** conceptualization (equal), data curation (equal), formal analysis (equal), investigation (equal), methodology (equal), writing – original draft (supporting). **Zhihong Qian:** data curation (supporting), writing – original draft (supporting), writing – review and editing (supporting). **Xiaoyun Zhou:** data curation (supporting), investigation (supporting), writing – original draft (supporting). **Zhaohui Zhu:** conceptualization (equal), funding acquisition (equal), methodology (equal), resources (equal), supervision (equal), validation (equal), writing – review and editing (supporting). **Yeye Chen:** conceptualization (equal), funding acquisition (equal), methodology (equal), resources (lead), supervision (equal), validation (equal), writing – original draft (supporting), writing – review and editing (equal).

## Ethics Statement

The study was approved by the institutional review board of the Peking Union Medical College Hospital (ZS1113).

## Consent

The patient wrote informed consent for examination and provided written informed consent for the publication of this report.

## Conflicts of Interest

The authors declare no conflicts of interest.

## Data Availability

The data that support the findings of this study are available from the corresponding author upon reasonable request.
